# Comparative Analysis of Chitosan, Lipid Nanoparticles, and Alum Adjuvants in Recombinant SARS-CoV-2 Vaccine: An Evaluation of Their Immunogenicity and Serological Efficacy

**DOI:** 10.3390/vaccines13080788

**Published:** 2025-07-24

**Authors:** Majed Ghattas, Garima Dwivedi, Anik Chevrier, Trevor Scobey, Rakan El-Mayta, Melissa D. Mattocks, Dong Wang, Marc Lavertu, Mohamad-Gabriel Alameh

**Affiliations:** 1 Institute of Biomedical Engineering, Polytechnique Montreal, Montreal, QC H3T 1J4, Canada; majed.ghattas@polymtl.ca; 2 Department of Chemical Engineering, Polytechnique Montreal, Montreal, QC H3T 1J4, Canada; anik.chevrier@polymtl.ca (A.C.); dong.wang@polymtl.ca (D.W.); 3 Department of Pathology and Lab Medicine, Perelman School of Medicine, University of Pennsylvania, Philadelphia, PA 19104, USA; garima.dwivedi@pennmedicine.upenn.edu; 4 Penn Institute for RNA Innovation, University of Pennsylvania, Philadelphia, PA 19104, USA; 5 Department of Epidemiology, University of North Carolina at Chapel Hill, Chapel Hill, NC 27599, USA; trevor.scobey@unc.edu; 6 Department of Bioengineering, University of Pennsylvania, Philadelphia, PA 19104, USA; elmayta1@seas.upenn.edu; 7 Department of Microbiology and Immunology, School of Medicine, University of North Carolina at Chapel Hill, Chapel Hill, NC 27599, USA; melissa_mattocks@med.unc.edu; 8 Raymond G. Perelman Center for Cellular and Molecular Therapeutics, Children Hospital of Philadelphia Research Institute, Philadelphia, PA 19104, USA; 9 Department of Experimental Pathology, Children Hospital of Philadelphia, Philadelphia, PA 19104, USA

**Keywords:** chitosan, adjuvant, vaccine, lipid nanoparticles, aluminum salts

## Abstract

**Background:** Chitosan, a family of polysaccharides composed of glucosamine and N-acetyl glucosamine, is a promising adjuvant candidate for eliciting potent immune response. **Methods:** This study compared the adjuvant effects of chitosan to those of empty lipid nanoparticles (eLNPs) and aluminum hydroxide (alum) following administration of recombinant SARS-CoV-2 spike immunogen in adult mice. Mice received the adjuvanted recombinant protein vaccine in a prime-boost regimen with four weeks interval. Subsequent analyses included serological assessment of antibody responses, evaluation of T cell activity, immune cell recruitment and cytokine profiles at injection site. **Results:** Compared to alum, chitosan induced a more balanced Th1/Th2 response, akin to that observed with eLNPs, demonstrating its ability to modulate both the humoral and cellular immune pathways. Chitosan induced a different proinflammatory cytokine (e.g., IL-1⍺, IL-2, IL-6, and IL-7) and chemokine (e.g., Eotaxin, IP-10, MIP-1a) profile compared to eLNPs and alum at the injection site and in the draining lymph nodes. Moreover, chitosan potentiated the recruitment of innate immune cells, with neutrophils accounting for about 40% of the infiltrating cells in the muscle, representing a ~10-fold increase compared to alum and a comparable level to eLNPs. **Conclusions:** These findings collectively indicate that chitosan has the potential to serve as an effective adjuvant, offering comparable, and potentially superior, properties to those of currently approved adjuvants.

## 1. Introduction

Vaccines effectively combat infectious diseases and save countless lives [1]. Global efforts to control the COVID-19 pandemic have underscored the urgency to develop effective and safe vaccines against severe acute respiratory syndrome coronavirus 2 (SARS-CoV-2). Recombinant protein-based vaccines represent one of the several platforms used for inducing protective immune responses against pathogens. The optimization of recombinant vaccine efficacy often requires the incorporation of adjuvants to promote potent and durable immune response [1]. Adjuvants play a crucial role in augmenting the immune response against antigens by inducing an environment that promotes antigen uptake and the activation of innate and adaptive immune pathways [2].

Chitosan, a natural polysaccharide derived from chitin by chemical or enzymatic deacetylation, has gained attention as a promising adjuvant for vaccine development [3,4,5]. The adjuvant capabilities of chitosan have been demonstrated to rely on the activation of the cytoplasmic DNA sensor cGAS and the STING pathway [3], along with the activation of the NLRP3 inflammasome [6], leading to the maturation of antigen-presenting cells (APCs), secretion of proinflammatory cytokines and chemokines, and the subsequent activation of cellular and humoral responses [3,5,7,8].

Lipid nanoparticles (LNPs) have garnered attention for their adjuvant abilities in vaccine development [9,10,11,12], in addition to their ability to encapsulate and deliver nucleic acids, such as messenger RNA, plasmid DNA, and small interfering RNAs. The adjuvant effect of LNPs relies mainly on the ionizable lipid, a major component of the formulation, and on the physico-chemical properties of the particles (e.g., size, shape, charge) with interleukin-1 beta (IL-1β) and IL-6 serving as key effector cytokines of the observed adjuvant effect [13]. For instance, specific cationic or ionizable lipids within LNPs have been demonstrated to activate Toll-like receptors (e.g., TLR-4) and induce the NLRP3 inflammasome pathway [13,14,15,16], triggering the release of proinflammatory cytokines/chemokines and the robust activation of humoral and cellular immune responses, including germinal center (GC)-B cell and T follicular helper (TfH) responses.

In contrast, aluminum salts (alum), including aluminum hydroxide and aluminum phosphate, have a long-standing history of use as vaccine adjuvants [17,18,19], primarily owing to their ability to induce T helper 2 (Th2) immune responses. The depot effect, defined as prolonged release of antigens, and once considered as alum’s main adjuvant mechanism [20], has since been widely questioned [21,22,23,24]. However, the precise mechanisms underlying the adjuvant effects of alum remain to be fully elucidated [25].

We believe that a thorough comparative evaluation of chitosan and known adjuvants such as LNPs and alum is needed to validate the use of chitosan as a viable adjuvant in humans. We hypothesized that chitosan-adjuvanted vaccines will induce high-avidity neutralizing antibodies and antigen-specific T-cell responses at levels comparable to, or better than, those elicited by eLNPs and alum. In addition, we hypothesized that chitosan would exhibit a distinct proinflammatory cytokine signature and innate immune cell recruitment profile at the injection site due to activation of distinct cellular signaling cascades. To test these hypotheses, we assessed antibody and immune cell responses following intramuscular administration of these adjuvants admixed to SARS-CoV2 spike recombinant protein. We also assessed cytokine levels and the magnitude and phenotypes of cells infiltrating the injection site and the draining lymph nodes (dLNs) in mice for each of the adjuvants tested.

## 2. Methods

### 2.1. High-Purity Chitosan

Raw chitosan (Marinard Biotech, Gaspé, QC, Canada) was heterogeneously deacetylated in concentrated NaOH to achieve the target degree of deacetylation (DDA) (92%) [26]. Nitrous acid was used to depolymerize chitosan, as previously described [26], to achieve a specific number-average molecular weight (Mn) target of 138 kDa. The resulting sample was freeze-dried and characterized for use in vaccine formulations. Chitosan with a 138 kDa Mn and 92% DDA was dissolved overnight on a rotary mixer at 0.5% (*w*/*v*) in hydrochloric acid using a glucosamine/HCl ratio of 1:1. The resulting solution was filter-sterilized through 0.2 μm filters (Millipore Sigma, Oakville, ON, Canada, Cat# SLGV004SL), aliquoted into smaller volumes, and stored at −80 °C until use.

### 2.2. Characterization of Chitosans

The number-average molecular weight (Mn) of chitosan was determined using size exclusion chromatography with multi-angle light scattering (SEC-MALS) (Supplementary Figure S1), following the protocol established by Nguyen et al. [27]

DDA was quantified using ^1^H NMR spectroscopy (Supplementary Figure S2) according to the method described by Lavertu et al. [28]. DDA was calculated using integrals of the peak of proton H1 of deacetylated monomer (H1-D) and of the peak of the three protons of the acetyl group (H-Ac): DDA (%) = $\left(\frac{H1D}{H1D+\frac{HAc}{3}}\right)\times 100$.

Endotoxin levels in chitosan samples were quantified using a kinetic chromogenic Limulus Amebocyte Lysate (LAL) Assay (Sensitivity 0.001 EU/mL) performed by Associates of Cape Cod, Inc. (East Falmouth, MA, USA).

Physicochemical characteristics of the chitosan are shown in Table 1 below.

### 2.3. Empty Lipid Nanoparticles and Aluminum Hydroxide

The LNP with no payload (empty LNPs, or eLNPs) formulation used in this study was composed of ALC-315, DSPC, cholesterol, DMG-PEG at a 50:10:38.5:1.5 mol ratio (%). eLNPs were prepared using an in-house microfluidic herringbone device, followed by ethanol removal and concentration using tangential flow filtration. The empty particles were characterized for their size (Z-average diameter) and polydispersity (~60 nm and 0.1) using dynamic light scattering (Malvern ZetaSizer™ Ultra, Malvern, UK), and their lipid content was determined to be 30 µg/µL. The eLNPs were stored at −80 °C until use.

Aluminum hydroxide gel, Alhydrogel^®^ 2%, referred to in this manuscript as alum, was purchased from InvivoGen (San Diego, CA, USA, Cat# 21645-51-2). The administered dose in all figures corresponds to the aluminum content.

### 2.4. Recombinant Protein

The SARS-CoV-2 spike recombinant protein used in the vaccine formulations was acquired from Sino Biological (Chesterbrook, PA, USA, Cat# 40589-V08H4). The S1 domain of the SARS-CoV-2 spike protein used in the ELISA was procured from GenScript (Piscataway, NJ, USA, Cat# Z03501).

### 2.5. Mice

Six- to eight-week-old female C57BL/6 or BALB/c mice were obtained from the Jackson Laboratory and Charles River, respectively, and housed in a conventional animal facility. All experiments in this study exclusively utilized female mice, which were randomly allocated to experimental groups. This study adhered to the guidelines outlined in the “Guide for the Care and Use of Laboratory Animals” by the National Research Council’s Committee on the Care of Laboratory Animal Resources. The animal procedures were reviewed and approved by the Institutional Animal Care and Use Committee (IACUC) at the University of Pennsylvania (protocol IACUC-803941). The mice were housed and maintained in an AAALAC-accredited facility, ensuring compliance with all applicable local, state, and federal regulations.

### 2.6. Mouse Immunization

**Figure 1:**
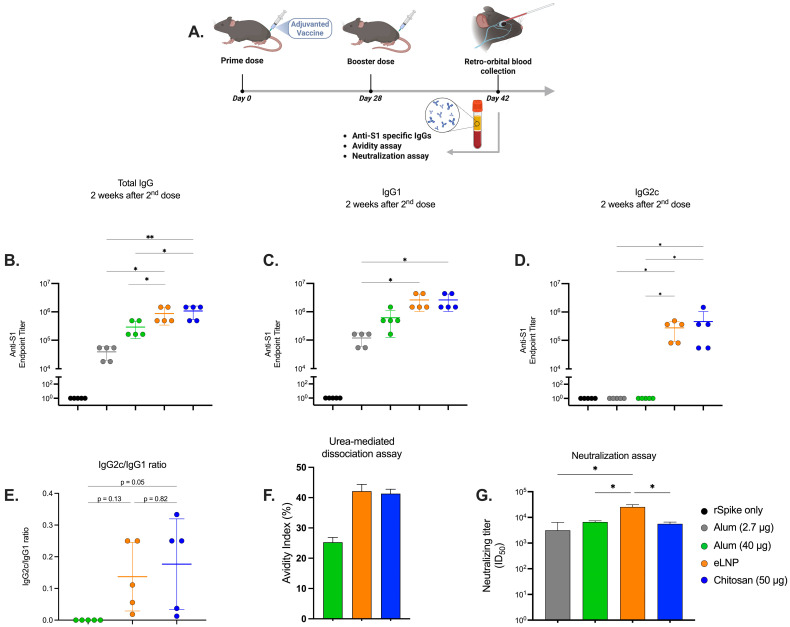
Humoral immune response induced by alum-, eLNP-, or chitosan-adjuvanted SARS-CoV-2 recombinant vaccines. (**A**) Experimental design of mice immunized with the adjuvanted recombinant SARS-CoV-2 vaccine. Serum levels of S1-specific IgG, IgG1, and IgG2c antibodies in vaccinated mice on day 14 post-boost are shown in (**B**), (**C**), and (**D**), respectively. Ratios of the indicated IgG subclasses in the vaccinated mice serum are shown in (**E**). Dissociation of low-affinity S1-specific IgGs was performed using 4 M urea, and the avidity index in the endpoint titer is presented in (**F**). Neutralizing titers were measured using a live virus neutralization assay (**G**). Results from two independent experiments. The group receiving eLNPs was administered at a dose equivalent to 3 µg of mRNA-LNP (this dose is equivalent to 90 µg of total lipid, or 45 µg of ionizable lipid). The control group received 5 µg of unadjuvanted recombinant SARS-CoV-2 protein. Data are presented as the mean ± SD (n = 5). *p*-values: * denotes *p* < 0.05, and ** *p* < 0.01.

Vaccines were administered via the intramuscular (IM) route into the gastrocnemius muscle, in 50 μL total volume, using a 3/10cc 29½G insulin syringe (Cardinal Health, Dublin, OH, USA, Cat# 8881600145). All immunizations were performed under anesthesia using isoflurane vaporizer. For the chitosan injections, 7% *w*/*v* sucrose in 25 mM 2-(*N*-morpholino) ethanesulfonic acid (MES) buffer with pH 6 was used as an injection solution. For the eLNP and alum injections, PBS was used as injection solution. Mice (n = 5) received immunizations using a prime-boost strategy at a 4-week interval. Mice were IM immunized with 5 μg dose of the SARS-CoV-2 S recombinant protein adjuvanted with 50 μg chitosan and compared to a similar dose of recombinant protein admixed with eLNPs at an mRNA equivalent dose of 3 µg (~90 µg total lipid content or 45 µg ionizable lipid content) or 2.7 μg alum (positive charge equivalent dose to chitosan) (Figure 1A). An additional dose of 40 µg alum was tested to mimic the equivalent dose administered to humans [29]. The control group received an unadjuvanted dose of 5 μg dose of the SARS-CoV-2 S recombinant only.

### 2.7. Blood Collection

Mice were anesthetized with isoflurane vaporizer, and blood was collected through the retro-orbital route into SST tubes (BD Microtainer^®^, San Jose, CA, USA, Cat# 365967) two weeks after the boost vaccination. Following incubation of 30 min at room temperature, the tubes were centrifuged at 10,000× *g* for 5 min at room temperature. The separated serum was transferred into 1.5 mL tubes and stored at −20 °C until use.

### 2.8. Determination of Anti-S1 Antibody Titers Using Endpoint Titer Enzyme-Linked Immunosorbent Assay (ELISA)

High Bind Stripwell™ Corning 96-Well Clear Polystyrene Microplates were prepared by coating them overnight at 4 °C with 1 μg/mL of purified His-tagged SARS-CoV-2 S1 protein. The plates were washed once with a wash buffer containing 0.05% Tween-20 (Bio-Rad, Saint-Laurent, QC, Canada, Cat# 1706531) in PBS. To block nonspecific binding sites, the plates were incubated with a solution of 2% (*w*/*v*) heat-inactivated, IgG-depleted, protease-free bovine serum albumin (BSA) (Sigma-Aldrich, St-Louis, MO, USA, Cat# A7030-500G) in PBS at room temperature for 2 h. After blocking, the plates were washed three times with wash buffer. Serial dilutions of C57BL/6 mouse sera were then prepared in the blocking solution in the plates and incubated for 2 h at room temperature. Following another three washes, HRP-conjugated anti-mouse secondary antibodies specific to total IgG (Abcam, Cambridge, UK, Cat#97040) (1:10,000) or subclasses (IgG1 (Abcam, Cambridge, UK, Cat# 98693) and IgG2c (Abcam, Cambridge, UK, Cat# 98722), each at 1:10,000) diluted in the blocking buffer were added. The plates were incubated for 1.5 h at room temperature and then washed three times. TMB substrate from KPL (Avantor, Radnor, PA, USA, Cat# 95059-156) was added to the wells and allowed to react for 8 min. The enzymatic reaction was halted by adding 2N sulfuric acid, and the absorbance was read at 450 nm using a Varioskan™ LUX Multimode microplate reader (Thermo Fisher Scientific, Waltham, MA, USA). The S1-specific antibody end-point dilution titer was defined as the highest dilution of serum to yield an optical density (OD) greater than the cut-off OD value determined using the Frey method [30].

### 2.9. Urea-Mediated Dissociation of Low-Avidity SARS-CoV-2 S1 Specific Antibodies

The serological detection of anti-SARS-CoV-2 S1 antibody titers was performed using endpoint ELISA. Prior to the addition of the HRP-conjugated secondary antibody, a chaotropic agent solution, 4M urea (Fisher Scientific, Waltham, MA, USA, Cat# BP169500) dissociation solution was added for 20 min [31], and the control groups were incubated with 2% BSA solution. The plates were then washed three times with wash buffer. The avidity index was estimated by calculating the titer of antibodies that remain bound to the antigen following treatment with the chaotropic agent:

$$Avidity\;Index\;\left(\%\right)=\frac{Titer\;with\;4M\;Urea}{Titer\;without\;4M\;Urea}\times 100$$

### 2.10. T-Cell Stimulation Assay and Intracellular Cell Staining

The animals were euthanized two weeks after the boost vaccination, according to the National Institutes of Health (NIH) Animal Research Advisory Committee Guidelines for Euthanasia of Rodents, using a Carbon Dioxide fill rate of 30–70% of the chamber volume per minute (4 min total), followed by cervical dislocation. The mice were then washed with ethanol, and the draining inguinal and popliteal lymph nodes were harvested, homogenized mechanically by rupturing between the frosted edges of microscope slides, and then passed through a 70 μm cell strainer in RPMI-1640 media (ATCC, Manassas, VA, USA, Cat# 30-2001) with 10% vol/vol heat inactivated fetal bovine serum (FBS) (Sigma-Aldrich, St-Louis, MO, USA, Cat# A7030-500G) and 1× penicillin–streptomycin (Gibco, Waltham, MA, USA, Cat# 15140122). All staining procedures were completed at 4 °C in FACS buffer (PBS with 2% vol/vol heat-inactivated FBS).

To evaluate antigen-specific T cells, 2 × 10^6^ cells were stimulated with SARS-CoV-2 spike peptide pools (JPT PM-WCPV-S) in a FACS tube for 6 h at 37 °C, 5% CO_2_, in the presence of 1 μg/mL anti-CD28 (Biolegend, San Diego, CA, USA, Cat# 102101) and 1 μg/mL anti-CD49d (Biolegend, San Diego, CA, USA, Cat# 103701). After 1 h of stimulation, 5 mg/mL of brefeldin A (Biolegend, San Diego, CA, USA, Cat# 420601), 2 mM of monensin (Biolegend, San Diego, CA, USA, Cat# 420701), and 5 mg/mL of anti-CD107a FITC were added for 5 h of additional stimulation. The mixture of 50 mg/mL phorbol 12-myristate 13-acetate and 1 mg/mL ionomycin served as the positive control, while DMSO served as the negative control. The cells were washed with FACS buffer, stained with viability stain (live/dead™, Thermo Fisher Scientific, Waltham, MA, USA, Cat# L34966) and anti-CD16/CD32 (Biolegend, San Diego, CA, USA, Cat# 101320) for Fc blocking. The cells were washed, fixed, and permeabilized with BD Cytofix/Cytoperm™ (BD, San Jose, CA, USA, Cat# 554714) solution before intracellular staining with a cocktail of antibodies in the dark at 4 °C. The samples were washed and fixed with 1% paraformaldehyde and acquired on a BD LSR-II Flow Cytometer. The gating strategy, as well as the antibody list and catalog numbers, are provided in Supplementary Figure S5 and Supplementary Table S1.

### 2.11. Protein Extraction from Muscle and Lymph Nodes

Mice (n = 4) received the same vaccine doses as detailed in the “Mouse immunization” section. Hind-limb muscles and lymph nodes were harvested at 6, 24, and 48 h post-injection from BALB/c mice, rapidly frozen in a mixture of isopropanol and dry ice, and stored at −80 °C until analysis. Frozen tissues were cut into small pieces on dry ice, weighed, and mechanically disrupted using the TissueLyzer^®^ II system (Qiagen, Toronto, ON, Canada). Disruption parameters differed by tissue type: the lymph nodes were processed at 2 × 30 Hz for 20 s per cycle, while muscles were processed at 4 × 30 Hz for 1 min per cycle, both using 5 mm steel beads (Qiagen, Toronto, ON, Canada, Cat# 69989). The homogenized tissues were then resuspended in 750 μL of M-Per lysis buffer (Thermo Fisher Scientific, Waltham, MA, USA, Cat# 78501) for lymph nodes or T-Per lysis buffer (Thermo Fisher Scientific, Waltham, MA, USA, Cat# 78510) for muscles, with both buffers supplemented with CoMplete protease inhibitor cocktail™ (Sigma-Aldrich, Oakville, ON, Canada, Cat# 11697498001). The samples were incubated on ice for 30 min, with gentle mixing every 10 min. The lysates were clarified by centrifugation at 2000× *g* for 30 min at 4 °C, after which the supernatants were transferred to fresh tubes and stored at −80 °C. A portion of each lysate was diluted 1:100 in PBS, and the total protein concentration was determined using the Pierce microBCA protein assay kit™ (Thermo Fisher Scientific, Waltham, MA, USA, Cat# 23235). All samples were adjusted to a final volume of 300 µL and an equal total protein content prior to cytokine quantification.

### 2.12. Determination of Cytokine Levels

Tissue lysates were collected from mice at 6, 24, and 48 h post-vaccination and analyzed for a panel of 32 proinflammatory cytokines (Sigma-Aldrich, Oakville, ON, Canada, Cat# MCYTOMAG-70K) using Luminex^®^ technology. The assay plates were designed using the Milliplex^®^ assay builder and underwent quality control as per the manufacturer’s specifications. All assays were conducted by Eve Technologies Corp. (Calgary, AB, Canada). All samples were normalized based on total protein content, and the signal from the PBS control was subtracted from all measurements.

### 2.13. Single-Cell Isolation from Muscle Tissue

Mice were administered the same vaccine doses as described in the “Mouse immunization” section. At 6, 24, and 72 h post-injection, BALB/c mice were anesthetized using an isoflurane vaporizer, and whole-body perfusion was performed by injecting approximately 20 mL of perfusion buffer (0.5 mM EDTA in Ca^2+^ and Mg^2+^-free PBS) into the left ventricle.

After perfusion, hind limb muscles were carefully dissected, ensuring the bones remained intact to prevent contamination. The sciatic nerve, connective tissue, popliteal lymph node, and excess fat were removed from the skeletal muscle. The muscle was weighed, minced into a uniform consistency, and digested in a medium containing 100 U/mL Collagenase IV (Thermo Fisher Scientific, Waltham, MA, USA, Cat# 17104-019) and 1.1 U/mL Dispase II (Roche, Indianapolis, IN, USA, Cat# 54905400) in Ham’s F-12 Nutrient Mix (Thermo Fisher Scientific, Cat# 11765054) for 1 h at 37 °C. Following digestion, the muscle cell suspension was passed through a 70 µm cell strainer (Falcon, Fisher Scientific, Waltham, MA, USA, Cat# 352350), centrifuged at 400× *g* for 5 min, and resuspended in 500 µL of DMEM/F12 medium (Thermo Fisher Scientific, Waltham, MA, USA, Cat# 10565042). The staining antibody list and catalog numbers are provided in Supplementary Table S2.

### 2.14. Live Virus Preparation and Neutralization Assay

Live recombinant SARS-CoV-2-nanoLuc virus (rSARS-CoV-2-nLuc) was prepared as described before [32], and serum neutralization experiments were performed under BSL-3 conditions. Vero E6 cells were seeded at 2 × 10^4^ cells/well in a 96-well plate 24 h prior to the neutralization assay. Serum samples were diluted 1:50 and subjected to a 3-fold eight-point dilution curve. Seventy-five plaque-forming units (PFUs) of rSARS-CoV-2-nLuc were mixed with diluted serum at a 1:1 ratio and incubated at 37 °C for 1 h; after that, the virus was added to cells and incubated at 37 °C in 5% CO_2_ for 48 h. Luciferase was measured as relative luminescence units (RLUs) by a Nano-Glo luciferase assay system (Promega, Madison, WI, USA, Cat# N1110) following manufacturer protocols using a SpectraMax M3 luminometer (Molecular Devices, San Jose, CA, USA). Percent neutralization was calculated using the following equation: 1 − $\left(\frac{RLU\;with\;sample}{RLU\;with\;mock\;treatment}\right)\times 100$.

SARS-CoV-2 neutralization titers (ID50) were defined as the sample dilution at which a 50% reduction in RLU was observed relative to the average of the virus control wells.

### 2.15. Statistical Analysis

Data are presented as mean ± SD. To compare either two or multiple groups, a Student’s *t*-test or one-way analysis of variance (ANOVA) followed by post hoc Tukey test, respectively, were utilized. *p* < 0.05 was considered to be statistically significant.

## 3. Results

### 3.1. Chitosan Induces an Effective Humoral Response in Mice

To assess the humoral immune response induced by chitosan-adjuvanted vaccines compared to eLNPs and alum, endpoint ELISA was conducted on serum samples collected from vaccinated mice. The serological analysis revealed a significant increase in IgG and IgG2c titers for chitosan and eLNPs in comparison to both alum doses (2.7 and 40 μg) (Figure 1B,D) and higher IgG1 titers compared to the 2.7 μg dose of alum (Figure 1C). The antibody titers for chitosan were comparable to those for eLNPs for all serotypes and superior to those of alum. In addition, chitosan and eLNPs displayed a higher IgG2c/IgG1 ratio compared to alum, indicating that both chitosan and eLNPs tend to trigger Th1-biased responses (Figure 1E). Since differences in antibody quality are essential for the efficacy of vaccines, we investigated antibody avidity using an urea-mediated dissociation assay. In this assay, a chaotropic agent is incubated with the antigen-antibody complexes to disrupt low affinity/avidity interactions, therefore allowing the estimation of the percentage of antibodies with high affinity/avidity by comparing signals in samples treated with the chaotropic agents versus untreated samples. The alum adjuvant showed an avidity index of 24% compared with 45% and 43% for eLNPs and chitosan respectively (Figure 1F) suggesting that both chitosan and eLNPs generate higher affinity/avidity antibodies. We tested the potential of these antibodies to neutralize live SARS-CoV-2 virus. Neutralizing antibody titers (ID_50_) were measured. The eLNP-adjuvanted vaccine elicited significantly higher neutralizing titers compared to all other groups. The chitosan-adjuvanted vaccine induced neutralization titers that were comparable to those observed with alum (Figure 1G). All adjuvanted formulations were well tolerated in mice, as evidenced by stable body weight throughout the study period (Supplementary Figure S3).

### 3.2. Chitosan Activates the Cellular Immune Response in Mice

**Figure 2:**
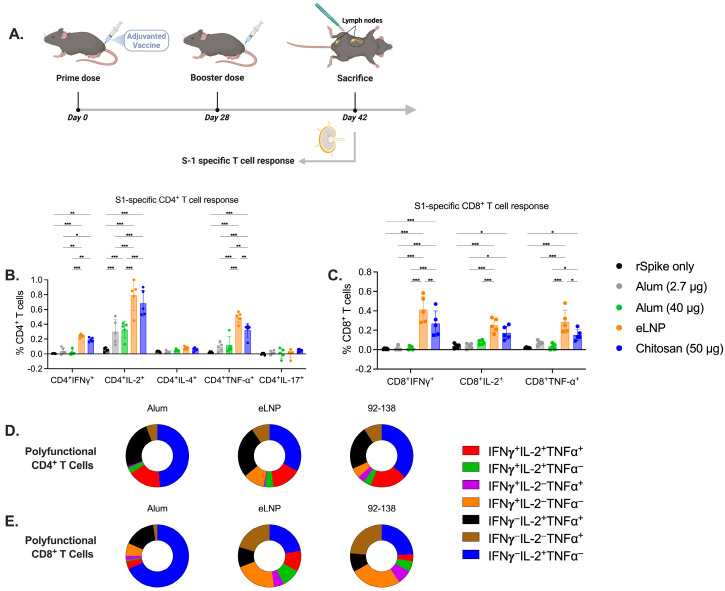
Cellular immune responses induced by alum-, eLNP- or chitosan-adjuvanted SARS-CoV-2 recombinant vaccine. (**A**) Experimental design of mice immunized with the adjuvanted recombinant SARS-CoV-2 vaccine. S1-specific CD4^+^ and CD8^+^ T-cell responses were evaluated by flow cytometry (**B**,**C**). T cells were labeled with cellular markers and assessed for the intracellular cytokine expression of Th1 (IFN-γ, IL-2, and TNF-α), Th2 (IL-4), and Th17 (IL-17a). (**D**,**E**) Pie charts representing the percentage of CD4^+^ (**D**) and CD8^+^ (**E**) polyfunctional cytokine-producing cells positive for two or three cytokines. Results from two independent experiments. The group receiving eLNPs was administered a dose equivalent to 3 µg of mRNA-LNP (this dose is equivalent to 90 µg of total lipid, or 45 µg of ionizable lipid). The control group received 5 µg of unadjuvanted recombinant SARS-CoV-2 protein. Data are presented as the mean ± SD (n = 5). *p*-values: * denotes *p* < 0.05, ** *p* < 0.01, and *** *p* < 0.001.

To assess the adaptive cellular immune response elicited by chitosan-adjuvanted vaccines in comparison to those elicited by eLNPs and alum adjuvants, a T-cell stimulation assay was performed. Following the immunization schedule, inguinal and popliteal draining lymph nodes (dLNs) were collected from the vaccinated mice and assessed for antigen-specific T cells (Figure 2A). Both chitosan and eLNPs activated CD4^+^ T cells in an antigen-specific manner and induced the secretion of Th1 polarizing cytokines to a greater extent than both alum groups. However, no Th2 or Th17 cytokine-secreting cells were detected in any of the groups tested (Figure 2B). Chitosan and eLNPs showed greater activation and secretion of IFN-*γ* and TNF-⍺ cytokines by CD8^+^ T cells compared to alum, which exhibited minimal activation (Figure 2C). Furthermore, we analyzed the frequency of polyfunctional T cells and observed a significant difference between alum and eLNPs for IFN*γ*^−^IL-2^+^TNF-⍺^+^ CD4^+^ cells, with eLNPs inducing a higher percentage compared to alum, but similar to chitosan (Figure 2D). For CD8^+^ polyfunctional cells (Figure 2E), eLNPs exhibited a higher percentage of triple-positive cells compared to the other groups. Together, these findings suggest that chitosan induces a higher frequency of monofunctional antigen-specific CD4 and CD8 T cells, as well as a distinct polyfunctional T cell signature compared to the response induced by alum, and is comparable to eLNPs.

### 3.3. Chitosan Induces Proinflammatory Cytokine Release at the Injection Site and Draining Lymph Nodes, Different from Alum and eLNPs

**Figure 3:**
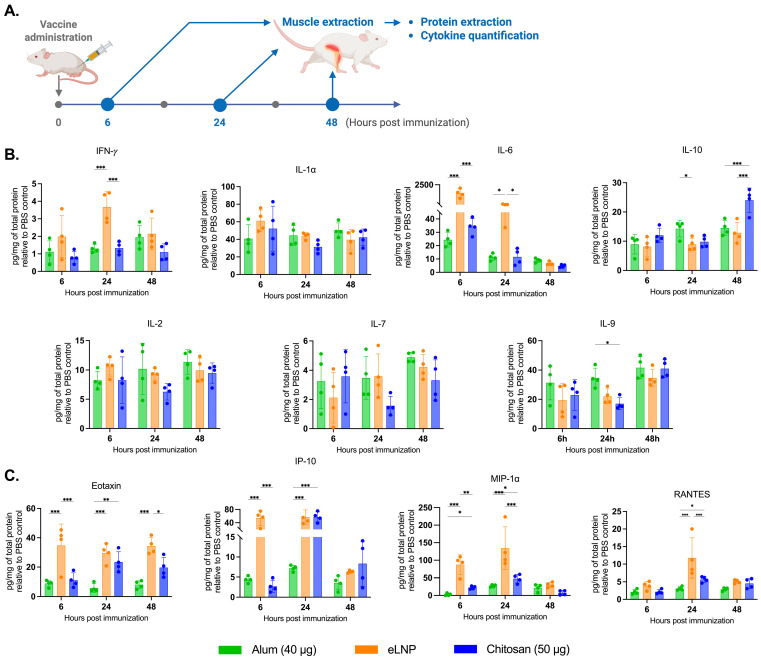
Proinflammatory cytokine and chemokine production in muscle tissue after vaccination. (**A**) Experimental design of the muscle tissue extraction in samples 6, 24, and 48 h after vaccination with alum-, eLNP-, or chitosan-adjuvanted recombinant SARS-CoV-2 vaccine. (**B**) Production of proinflammatory cytokines: IFN-*γ*, IL-1α, IL-6, IL-10, IL-2, IL-7 and IL-9. (**C**) Production of proinflammatory chemokines: Eotaxin, IP-10 (CXCL10), MIP-1α, and RANTES. The eLNP group was administered a dose equivalent to 3 µg of mRNA-LNP (this dose is equivalent to 90 µg of total lipid, or 45 µg of ionizable lipid). Data are presented as the mean ± SD (n = 4) relative to the control group (PBS injection). *p*-values: * denotes *p* < 0.05, ** *p* < 0.01, and *** *p* < 0.001.

**Figure 4:**
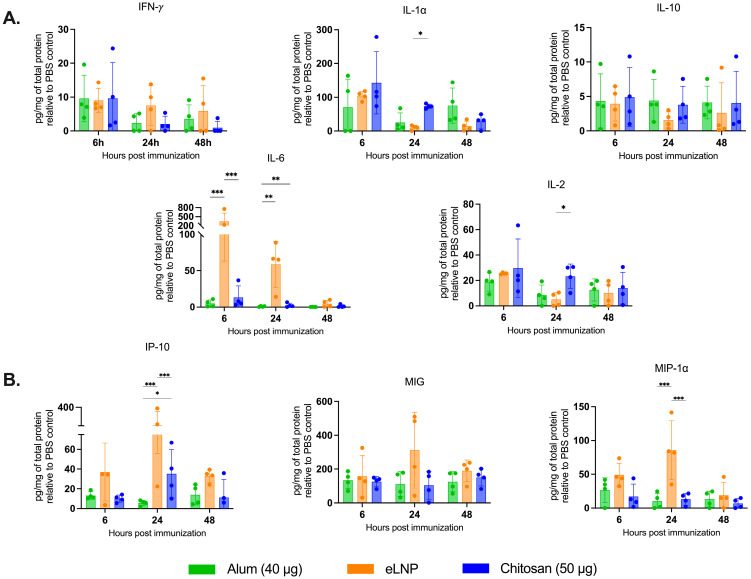
Proinflammatory cytokine and chemokine production in draining lymph nodes after vaccination. The samples were collected 6, 24 and 48 h after vaccination with alum- eLNP- or chitosan-adjuvanted recombinant SARS-CoV-2 vaccine. (**A**) Proinflammatory cytokines: IFN-*γ*, IL-1α, IL-10, IL-6 and IL-2 production. (**B**) Production of proinflammatory chemokines: IP-10 (CXCL10), MIG, and MIP-1α. The eLNP group was administered a dose equivalent to 3 µg of mRNA-LNP (this dose is equivalent to 90 µg of total lipid, or 45 µg of ionizable lipid). Data are presented as the mean ± SD (n = 4) relative to the control group (PBS injection). *p*-values: * denotes *p* < 0.05, ** *p* < 0.01, and *** *p* < 0.001.

Next, we measured the levels and profile of cytokines in tissue homogenates to investigate the immunostimulatory effect of chitosan at the injection site and in the dLNs, using a multiplex bead-based method (Figure 3A). Chitosan led to an increase in the levels of proinflammatory cytokines and chemokines (Figure 3) at the injection site and in the dLNs (Figure 4).

As expected, eLNPs significantly increased IL-6 expression in muscle at 6 and 24 h (1500 and 74 pg/mg respectively) (Figure 3B) and in the dLNs (340 and 60 pg/mg respectively) (Figure 4A). In contrast, chitosan induced a significant increase in IL-10 levels in the muscle at 48 h (24 pg/mg) compared to the other adjuvants (Figure 3B). In the dLNs, chitosan showed an increase in IL-1⍺ at 6 and 24 h (143 and 73 pg/mg) compared to eLNPs (104 and 10 pg/mg) and alum (67 and 20 pg/mg) (Figure 4A). Additionally, chitosan triggered a significant increase in proinflammatory chemokines (Eotaxin, IP-10, MIP-1α, and RANTES) after 24 h, surpassing the response observed with alum (Figure 4B). Altogether, these findings suggest that chitosan elicits a different cytokine response, or signature, compared to eLNPs, and alum.

### 3.4. Chitosan Potentiates Innate Immune Cell Recruitment at the Injection Site

**Figure 5:**
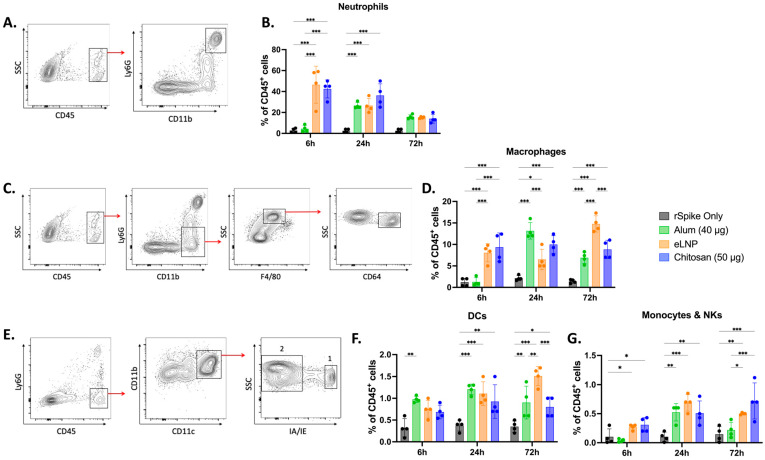
Innate immune cell recruitment induced by adjuvanted SARS-CoV-2 recombinant vaccine at the injection site. Mice were injected intramuscularly (IM) with recombinant SARS-CoV-2 vaccine adjuvanted with alum, eLNPs, or chitosan. Innate immune cell recruitment was examined at 6, 24, and 72 h in the gastrocnemius muscle by flow cytometry. (**A**,**B**) Representative flow cytometry gating strategy for neutrophils (CD45^+^Ly6G^+^CD11b^+^) (**A**) and their percentages (**B**). (**C**,**D**) representative gating strategy for macrophages (CD45^+^Ly6G^−^CD11b^+^F4/80^+^CD64^+^) (**C**) and their percentages (**D**). (**E**–**G**) Representative gating strategy for dendritic cells (1: CD45^+^Ly6G^−^CD11b^+^CD11c^+^IA/IE^+^), monocytes, and natural killer cells (2: CD45^+^Ly6G^−^CD11b^+^CD11c^+^IA/IE^−^) (**E**) and their percentages ((**F**) and (**G**), respectively). The eLNP group was administered a dose equivalent to 3 µg of mRNA-LNP (this dose is equivalent to 90 µg of total lipid, or 45 µg of ionizable lipid). The control group received 5 µg of unadjuvanted recombinant SARS-CoV-2 protein. Data are presented as the mean ± SD (n = 4). *p*-values: * denotes *p* < 0.05, ** *p* < 0.01, and *** *p* < 0.001.

Given the differences in cytokine/chemokine profiles between the different adjuvants tested, we examined and compared the potential of the three adjuvants to promote cell recruitment at the injection site. CD45^+^ immune cell recruitment to the muscle was observed in all groups (Supplementary Figure S4). Th chitosan-adjuvanted vaccines recruited 43 ± 8% neutrophils 6 h post-administration, ~10 times more than alum (5 ± 3%) and comparable to eLNPs (46 ± 18%) (Figure 5B). Macrophage recruitment was also higher with chitosan (9 ± 3%) and eLNPs (8 ± 2%) than with alum (1.0 ± 0.8%) at 6 h, but all groups showed similar levels (~10–15%) by 72 h (Figure 5D). The recruitment of dendritic cells (DCs) to the injection site was best achieved in the eLNP group (2.0 ± 0.1%) compared to the 2 other adjuvants (Figure 5F). Monocytes and natural killer cells showed no significant difference among the groups at early timepoints but exhibited some differences at 72 h after injection (Figure 5G).

## 4. Discussion

The development of effective vaccines relies on a thorough understanding of innate and adaptive immune responses. Adjuvants, which are immune-potentiating components of vaccine formulations enhance the magnitude and durability of the response to co-administered antigens [33]. Understanding the effect of adjuvants is crucial for designing safe vaccine formulations that elicit the desired adaptive immune response against targeted pathogens. This study investigated the adjuvant potential of chitosan, a natural biopolymer, when administered with a recombinant SARS-CoV-2 vaccine, and compared it to adjuvant activity observed with eLNPs and alum.

Our findings demonstrate that chitosan-adjuvanted vaccines elicit enhanced humoral and cellular immune responses compared to the traditional alum adjuvant. Chitosan-induced IgG and IgG2c titers were higher compared to those induced by alum, suggesting that chitosan can induce a more robust antibody production compared to alum and a similar response to eLNPs, which have been shown to exhibit strong adjuvant activity. Moreover, the IgG2c/IgG1 ratio showed that chitosan and eLNPs tend to induce Th1 polarization compared to that of alum, confirming their potential to evoke a more balanced antiviral and effective immune response [34].

The observed avidity and neutralizing potential of chitosan-induced antibodies support the capacity of chitosan to elicit high-affinity, functional antibodies capable of neutralizing the target antigen. This may be indicative of an efficient germinal center response and improved affinity maturation, two key processes in the development of durable and protective humoral immunity [35]. Compared to alum, chitosan demonstrated an enhanced ability to generate antibodies with superior avidity (Figure 1F). Despite an improved avidity index, both chitosan and alum displayed comparable neutralizing potential (Figure 1G). This may be associated with the fact that neutralization depends more on epitope specificity than on the total binding strength measured by the assay [36,37].

Chitosan-induced antibodies exhibited comparable titers and avidity to the eLNP-immunized mice (Figure 1F). However, eLNPs elicited a stronger neutralizing response compared to chitosan (Figure 1G). This significantly improved neutralization potential may be attributed to improved affinity maturation and/or an increased/more sustainable TfH response induced by increased IL-6 levels in the muscle and dLNs following eLNP administration (Figure 3B and Figure 4A) [12], or to improved DC recruitment (Figure 5F). IL-6 levels in mice are important drivers of affinity maturation and TfH responses [12,38,39]. In sum, our findings support our initial hypothesis that chitosan-adjuvanted vaccines can effectively induce antigen-specific, high-avidity neutralizing antibodies at levels comparable to, or superior to, those induced by some adjuvants such as alum.

In parallel, our evaluation of cellular immune responses revealed that both chitosan and eLNPs significantly enhanced the activation of CD4^+^ and CD8^+^ T lymphocytes compared to alum (Figure 2). This cellular response underscores the potential of chitosan in stimulating both arms of the adaptive immune response. The activation of CD8^+^ T cells is particularly important in the context of viral infections and intracellular pathogens, where cytotoxic responses play a central role in the clearance of infected cells [40]. In addition to conventional markers of T-cell activation, this study further assessed the induction of T cells capable of simultaneously producing multiple cytokines, which are increasingly recognized as key indicators of effective and long-lasting cellular immunity [41,42]. Specifically, the analysis focused on CD4^+^ and CD8^+^ T cells co-expressing IFN-γ, IL-2, and TNF-⍺. The results revealed a markedly higher proportion of IFN-γ^+^ IL-2^+^ TNF-⍺^+^ CD4^+^ T cells in animals immunized with chitosan and eLNP-adjuvanted vaccines compared to those receiving alum. For CD8^+^ T cells, eLNPs again showed the highest frequency of triple-positive cells, followed by chitosan, then alum. Taken together, these findings support our hypothesis that chitosan induces robust T-cell responses in the dLNs compared to the other adjuvants.

Cytokine profiling unveiled a proinflammatory cytokine response triggered by chitosan at both the injection site and dLNs, featuring elevated levels of Th1 cell response mediators (e.g., IL-1 and IP10) [34]. Interestingly, 48 h post-injection, the elevated levels of IL-10, a cytokine associated with Th2 response and recognized for its anti-inflammatory properties [43], suggest that chitosan effectively regulates immune responses. The subdued IL-6 response in muscle tissue with chitosan, compared to eLNPs, suggests an initiation of a moderate proinflammatory environment that could reduce clinical adverse events such as local pain [44,45] and lower systemic symptoms [46]. Building on the observed variations in cytokine and chemokine expression, we investigated how chitosan compares to eLNPs and alum in promoting innate immune cell recruitment. At the injection site, chitosan induced a strong early recruitment of neutrophils and macrophages at 6 h post-injection, showing a pattern of immune activation that aligns more closely with eLNPs than with alum. While alum exhibited a later peak in cell infiltration, and eLNPs showed delayed dendritic cell recruitment, chitosan was associated with more stable immune cell recruitment levels over time, suggesting distinct kinetic profiles among the adjuvants.

This observed variation in cytokine and immune cell recruitment profiles between chitosan and eLNPs suggests that distinct activation mechanisms may be at play for each adjuvant or material [47]. This difference highlights the possibility of a synergistic effect if these two components are combined, potentially enhancing immune response outcomes [48]. Innate immune cell recruitment studies further support the immunomodulatory properties of chitosan, demonstrating a significant increase in neutrophils, macrophages, and dendritic cells, particularly during the early phases after vaccination. This enhanced recruitment may be attributed to a depot effect where chitosan remains at the injection site for at least two weeks [49]. This indicates that chitosan not only enhances the immune response but also orchestrates a dynamic inflammatory environment, contributing to its overall effectiveness as a vaccine adjuvant.

## 5. Conclusions

In conclusion, chitosan can induce the production of proinflammatory cytokines and chemokines, potentiate innate immune cells, and elicit both humoral and cell-mediated immune responses, making it a promising intramuscular vaccine adjuvant platform. Its inherent biocompatibility and versatility through engineering the intrinsic properties of the polymer further enhance its potential as an alternative to conventional adjuvants.

## 6. Limitations

It is worth noting that although we observed robust neutralizing antibody responses using live virus assays, no viral challenge study was performed to directly assess the in vivo protective efficacy of the adjuvanted formulations. While neutralization titers are widely accepted as a correlate of protection for several viral infections, including SARS-CoV-2 [50], functional protection in animal model remains to be demonstrated. Second, our comparative analysis was limited to alum and eLNPs. While this provides useful benchmark comparisons, future work should include a more in-depth molecular analysis and a broader range of clinically used adjuvants to position chitosan’s performance within a more comprehensive immunological context. Third, different mouse strains were used in different experimental settings. For instance, C57BL/6 mice were employed for antibody quantification and T-cell analysis, whereas BALB/c mice were used for assessing muscle infiltration and local innate immune activation. Inter-strain immunological differences may influence the direct comparability of findings across datasets.

## Figures and Tables

**Table 1:** Physicochemical characteristics of the high-purity chitosan used in this study.

DDA (%) ^a^	Mn (kDa) ^b^	PdI	Endotoxin Content (EU/g) ^c^
92	138	1.31	24

^a^ Determined by ^1^H NMR. ^b^ Determined by SEC-MALS. ^c^ Determined by kinetic chromogenic Limulus Amebocyte Lysate (LAL) Assay.

## Data Availability

Data will be made available on request.

## Supplementary Materials

The following supporting information can be downloaded at: https://www.mdpi.com/article/10.3390/vaccines13080788/s1, Figure S1: Chitosan SEC-MALS chromatogram; Figure S2: Chitosan ^1^H NMR spectrum at 70 °C; Figure S3: Health assessment of mice over time after adjuvanted vaccines; Figure S4: CD45^+^ cell recruitment at injection site. Figure S5: Gating strategy for lymph node T cells; Table S1: Antibody staining panel for T cell activation assay; Table S2: Antibody staining panel for muscle cell infiltration assay.
