# Supplementary material for: Comparative Analysis of Chitosan, Lipid Nanoparticles, and Alum Adjuvants in Recombinant SARS-CoV-2 Vaccine: An Evaluation of Their Immunogenicity and Serological Efficacy
Source: Vaccines (Basel). 2025 Jul 24;13(8):788. doi: 10.3390/vaccines13080788 (PMC12390328; doi:10.3390/vaccines13080788)
Supplement: Supplementary file 1 [file vaccines-13-00788-s001.zip › vaccines-3320407-supplementary.pdf]

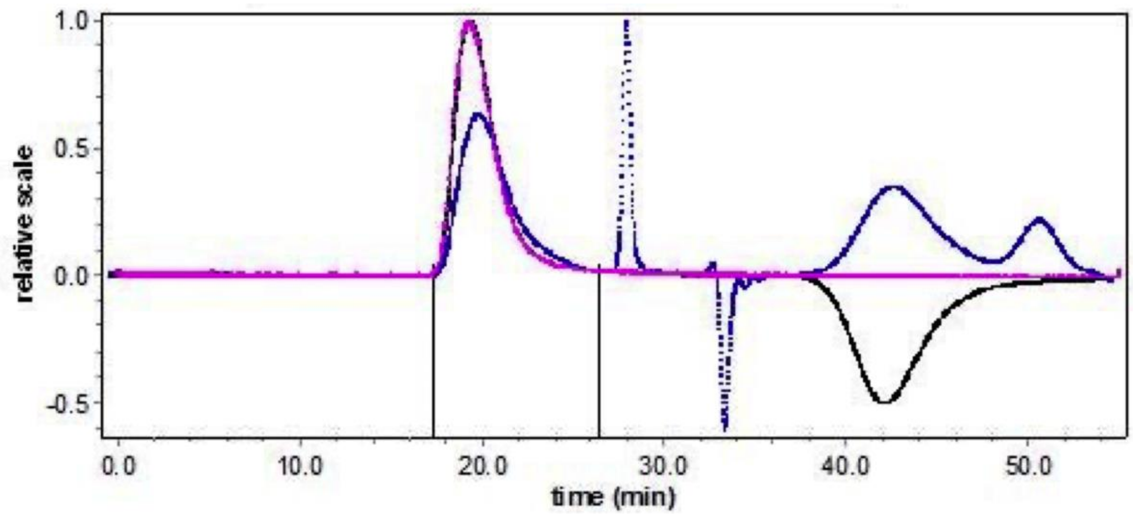

Supplementary Figure S1. Chitosan SEC-MALS chromatogram.

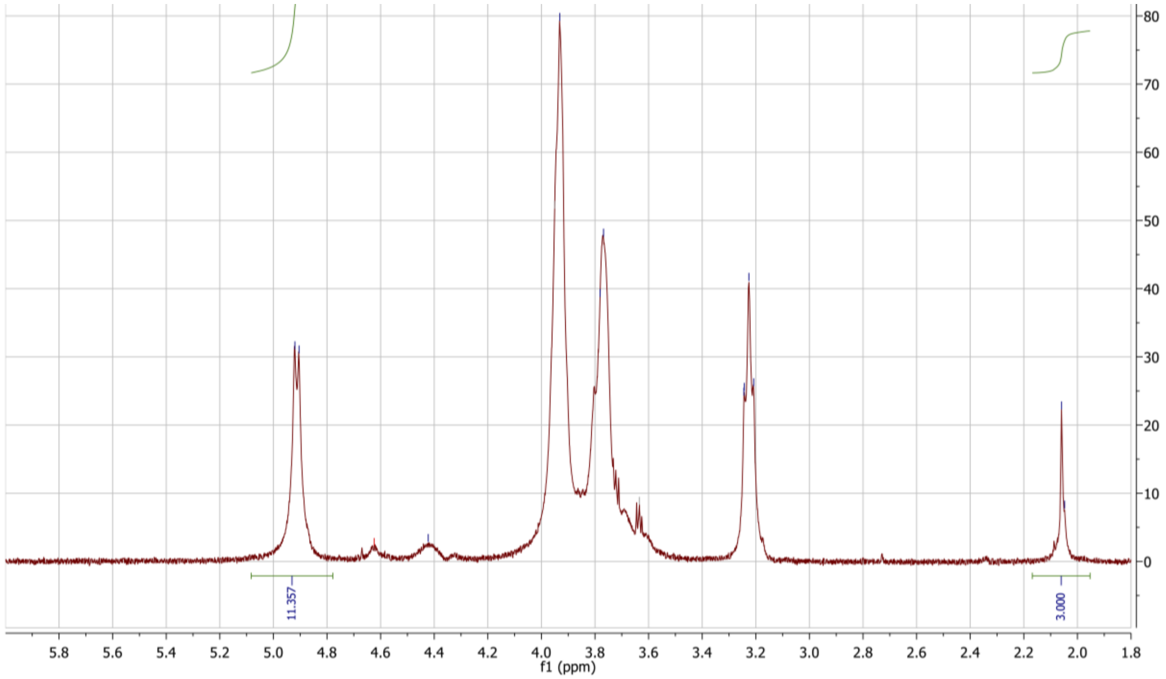

Supplementary Figure S2. Chitosan ^{1}H NMR spectrum at 70°C. The DDA was calculated using integrals of the peak of proton H1 of deacetylated monomer (H1-D) and of the peak of the three protons of acetyl group (H-Ac): DDA (%) = $\left(\frac{H1D}{H1D+\frac{HAc}{3}}\right) \times 100$

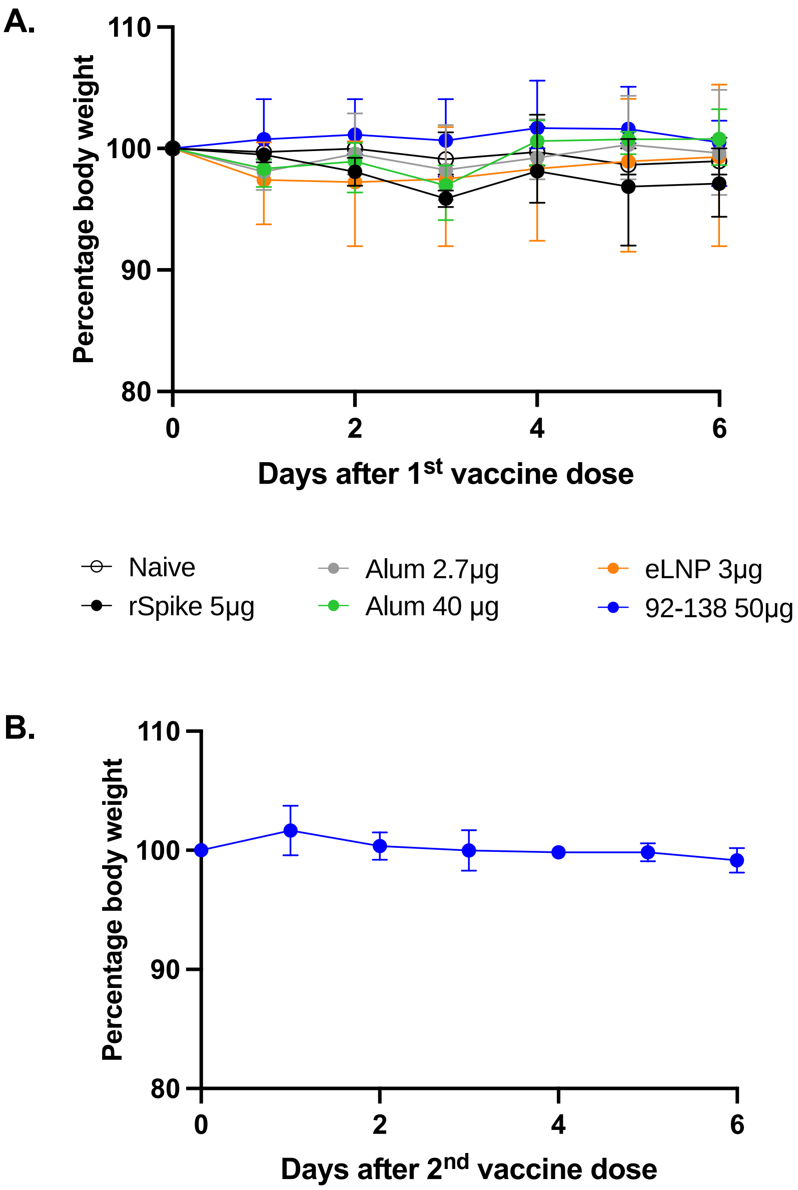

Supplementary Figure S3. Health assessment of mice over time after adjuvanted vaccines. Percentage bodyweight variation in mice (n=4) from different groups measured daily for one week following the administration of the first dose of the adjuvanted vaccine (A), and one week following the administration of the second dose of the chitosan (92-138) adjuvanted vaccine.

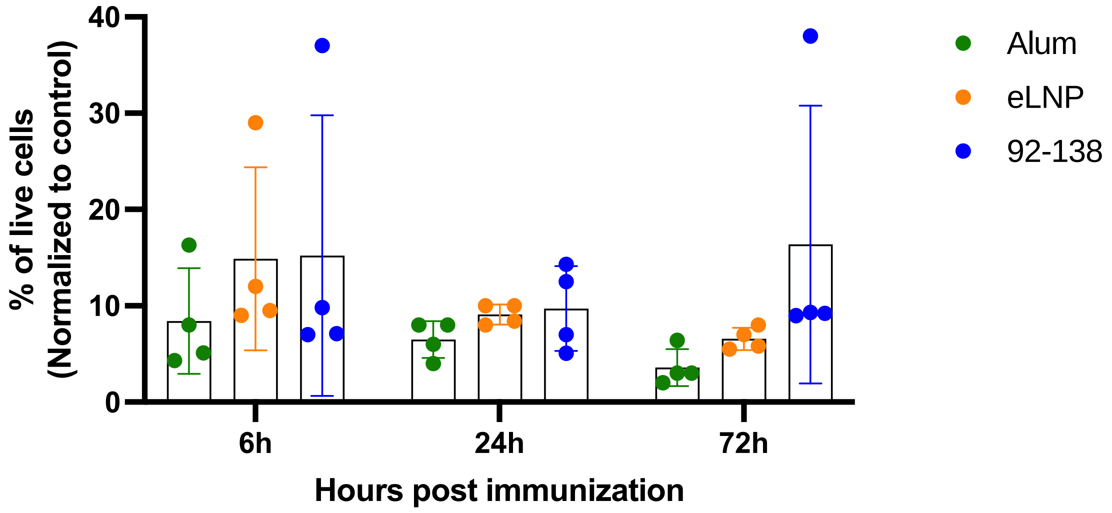

Supplementary Figure S4. CD45+ cell recruitment at injection site. The figure shows the percentage of live cells, normalized to control (PBS injection), expressing CD45 in the hindlimb muscle at 6-, 24-, or 72-hours post-vaccine administration, using different adjuvants. The data is presented as the mean ± standard deviation (SD) from four samples (n = 4) and is representative of two separate experiments.

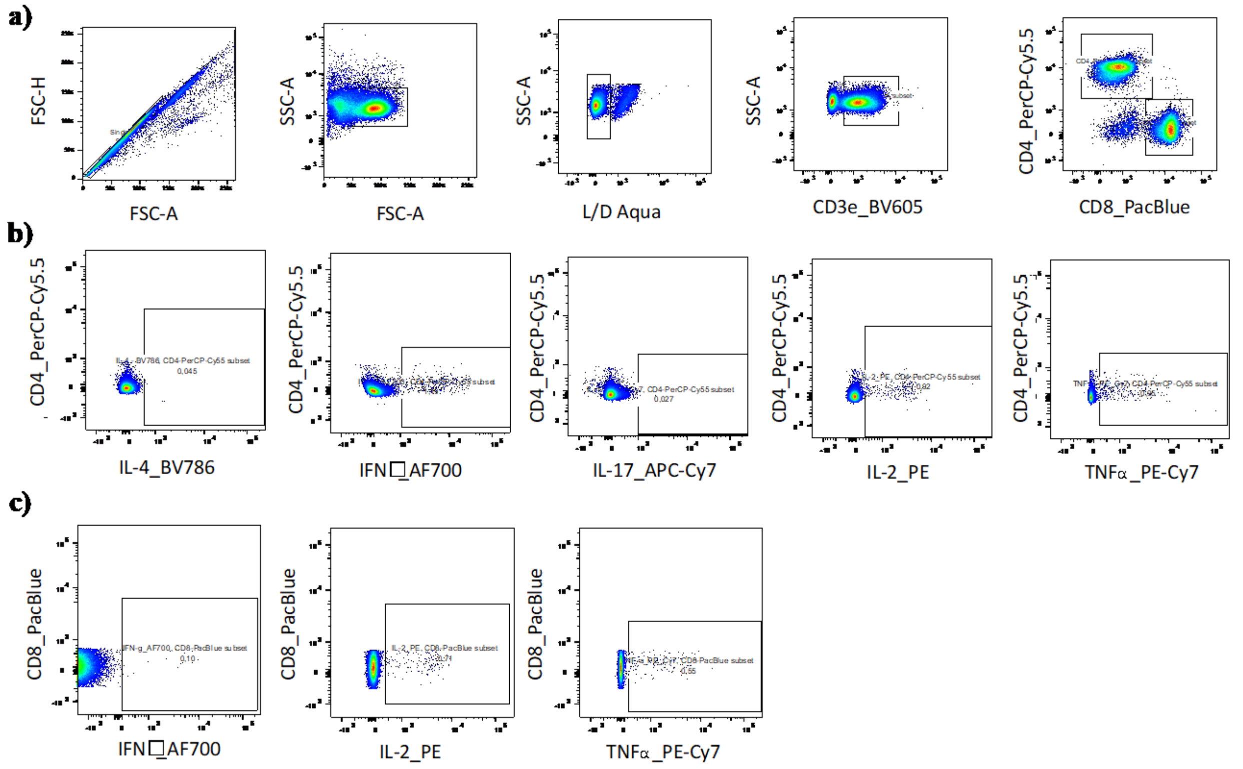

Supplementary Figure S5. Gating strategy for lymph node T cells. a) Gating scheme for the identification of CD4 + and CD8 + T cells. b) Identification of antigen specific cytokine-expressing CD4 + T cells. c) Identification of antigen specific cytokine-expressing CD8 + Tcells.

Supplementary Table S1: Antibody staining panel for T cell activation assay.

	Identifier	Source	Fluorochrome	Antibodies
	L34966	ThermoFisher	Aquablue	Live/Dead
**Cell surface markers staining**	100434	BioLegend	Peridinin Chlorophyll Protein-Cyanine5.5	CD4
	100725	BioLegend	Pacific Blue	CD8a
**Intracellular staining**	100351	BioLegend	Brilliant Violet 605	CD3e
	564006	BD Biosciences	Brilliant Violet 786	IL-4
	554429	BD Biosciences	Allophycocyanin	IL-2
	557998	BD Biosciences	Alexa Fluor 700	IFN-γ
	557644	BD Biosciences	Phycoerythrin/Cyanine7	TNF-α

Supplementary Table S2: Antibody staining panel for muscle cell infiltration assay.

**Identifier**	**Source**	**Fluorochrome**	**Antibodies**
127617	BioLegend	Phycoerythrin/Cyanine7	Ly6G
128005	BioLegend	Fluorescein isothiocyanate	Ly6C
101227	BioLegend	Peridinin Chlorophyll Protein-Cyanine5.5	CD11b
117321	BioLegend	Pacific Blue	CD11c
L34966	ThermoFisher	Aquablue	Live/Dead
107645	BioLegend	Brilliant Violet 785	IA/IE
103111	BioLegend	Allophycocyanin	CD45
123117	BioLegend	Allophycocyanin/Cyanine7	F4/80
139319	BioLegend	Phycoerythrin/Cyanine7	CD64
